# Supplementary material for: Contextual Constraints: Dynamic Evolution of Snake Venom Phospholipase A2
Source: Toxins (Basel). 2022 Jun 20;14(6):420. doi: 10.3390/toxins14060420 (PMC9231074; doi:10.3390/toxins14060420)
Supplement: Supplementary file 1 [file toxins-1732519-supplementary.pdf › toxins-1732519-supplementary.pdf]

Figure S1. Structural and electrostatic features of group I PLA_2s (Elapidae)

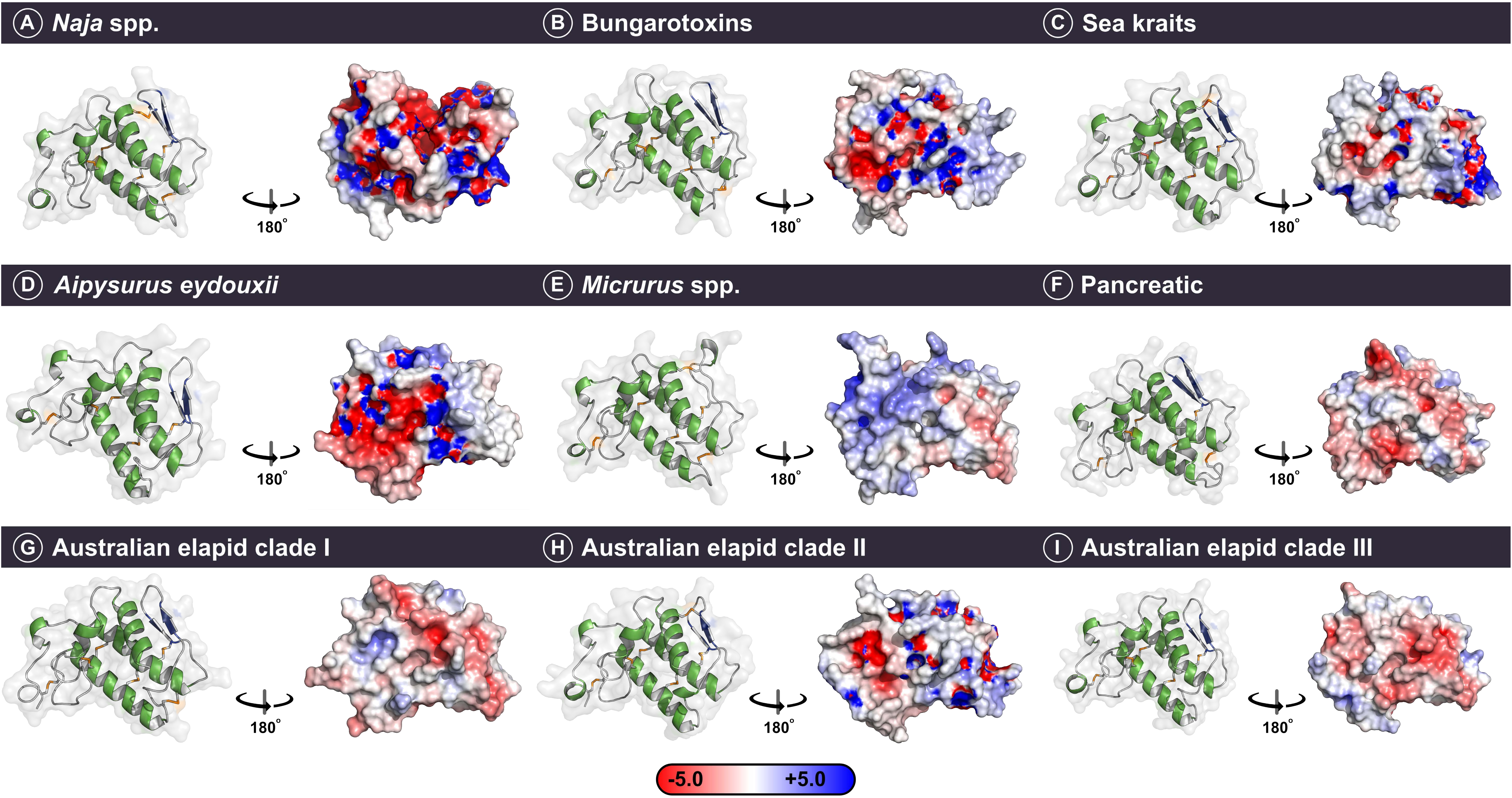

This figure illustrates the structural and electrostatic features of group I PLA_2s. Helices, sheets and disulfide bonds are shown in green, purple and orange colour, respectively. The surface electrostatic potential is shown on a graded scale from red (-5 units) to blue (+5 units).

Figure S2. Structural and electrostatic features of group II PLA₂s (Viperidae)

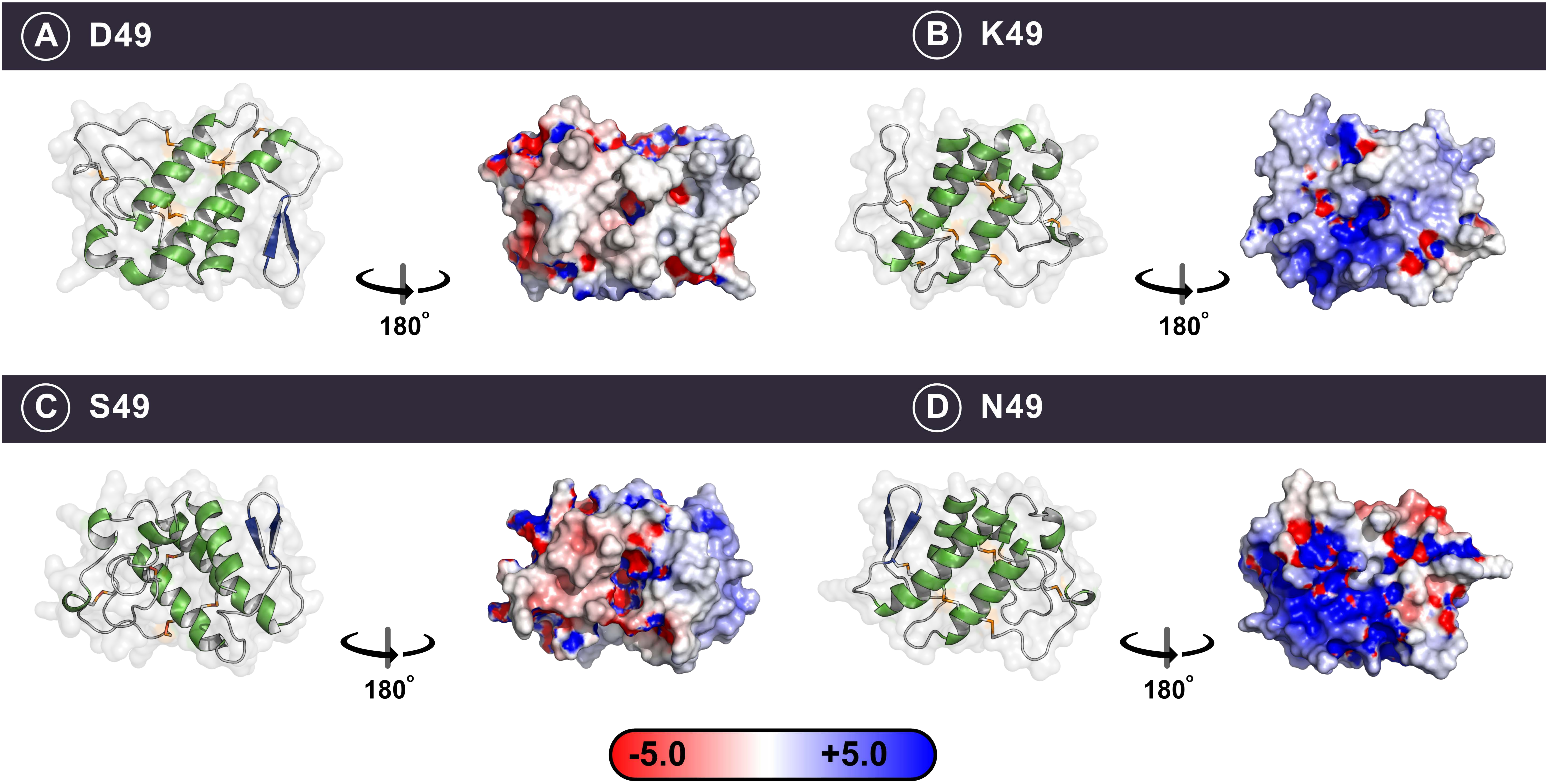

This figure illustrates the structural and electrostatic features of group II PLA_2s. Helices, sheets and disulfide bonds are shown in green, purple and orange colour, respectively. The surface electrostatic potential is shown on a graded scale from red (-5 units) to blue (+5 units).

Figure S3. Bayesian phylogenetic reconstruction of non-pancreatic group I PLA₂s (Elapidae)

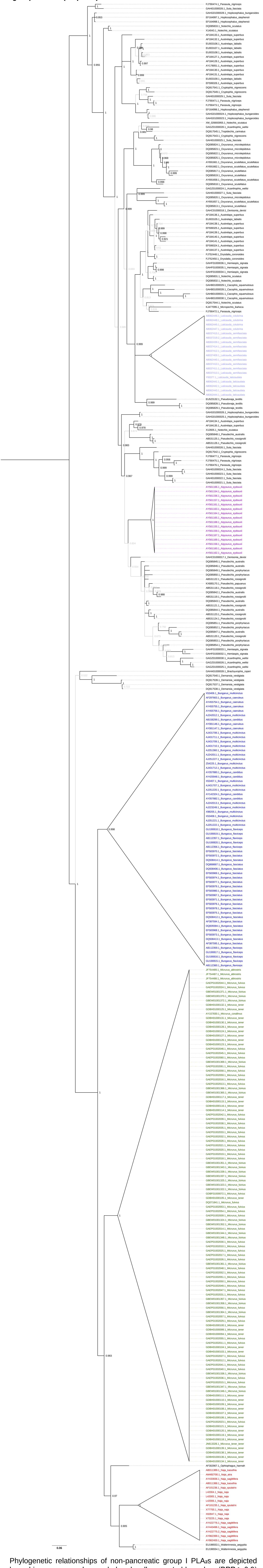

Phylogenetic relationships of non-pancreatic group I PLA₂s are depicted here. Lineages are colour coded and well-supported branches (BPP ≥ 0.9) are shown as thick black lines.

Figure S4. Bayesian phylogenetic reconstruction of pancreatic group I PLA_2s (Elapidae)

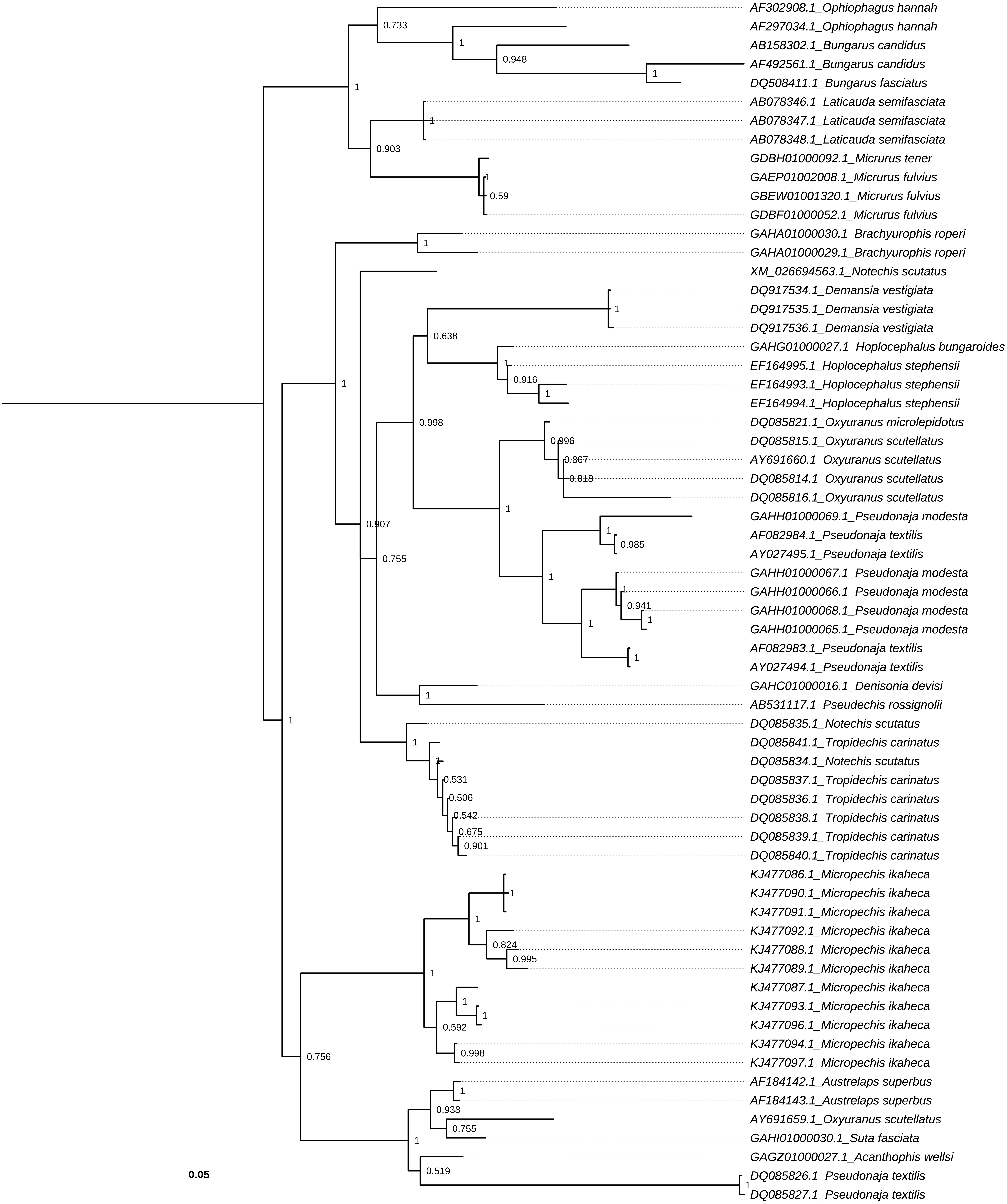

Phylogenetic relationships of pancreatic group I PLA_2s are depicted here. Thick black lines indicate well-supported branches (BPP ≥ 0.9).

Figure S5. Bayesian phylogenetic reconstruction for *Aipysurus* group PLA_2s

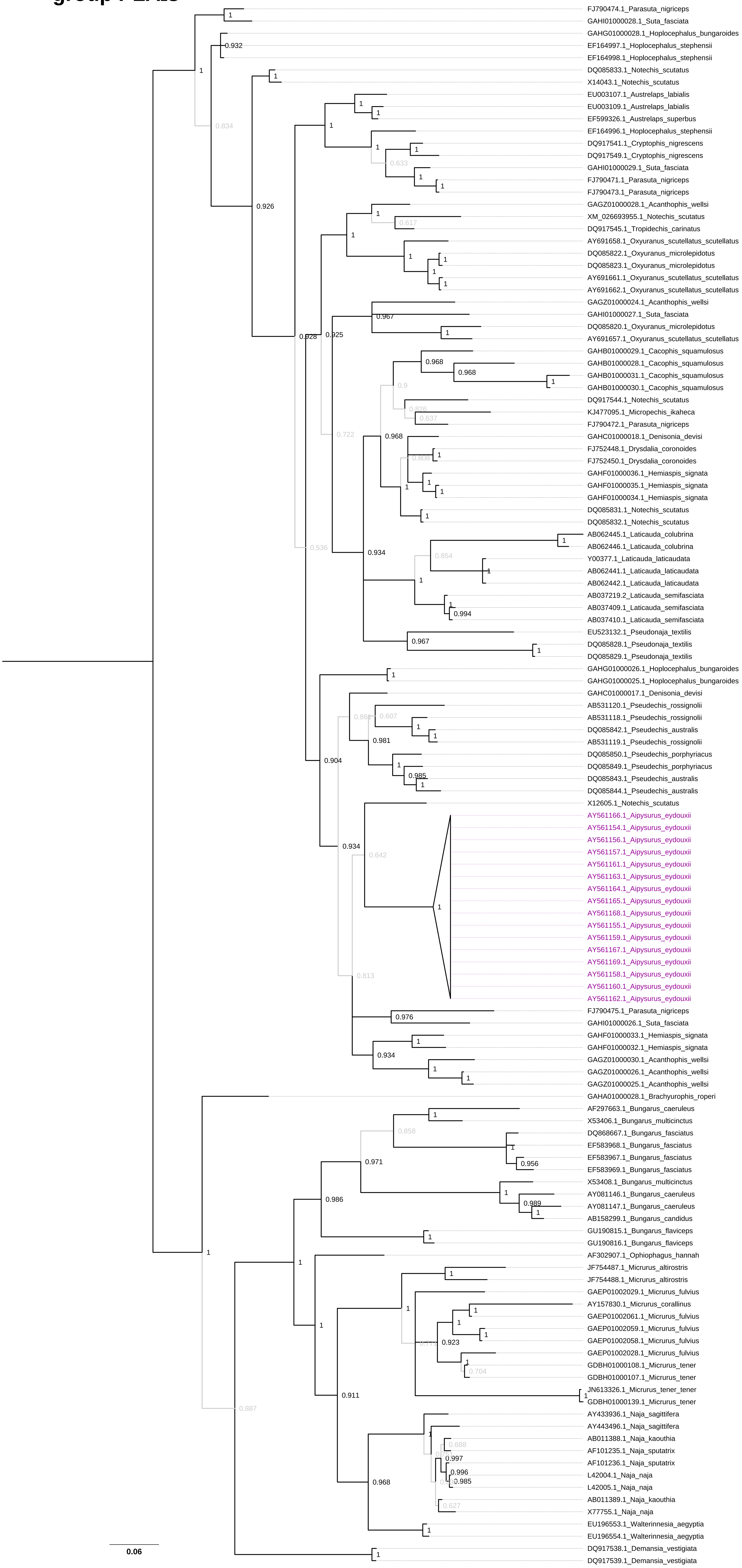

This figure portrays the phylogeny of the *Aipysurus* group with other non-pancreatic group I PLA_2 sets. Well-supported branches (BPP ≥ 0.9) are shown as thick black lines.

Figure S6. Bayesian phylogenetic reconstruction for β-bungarotoxins

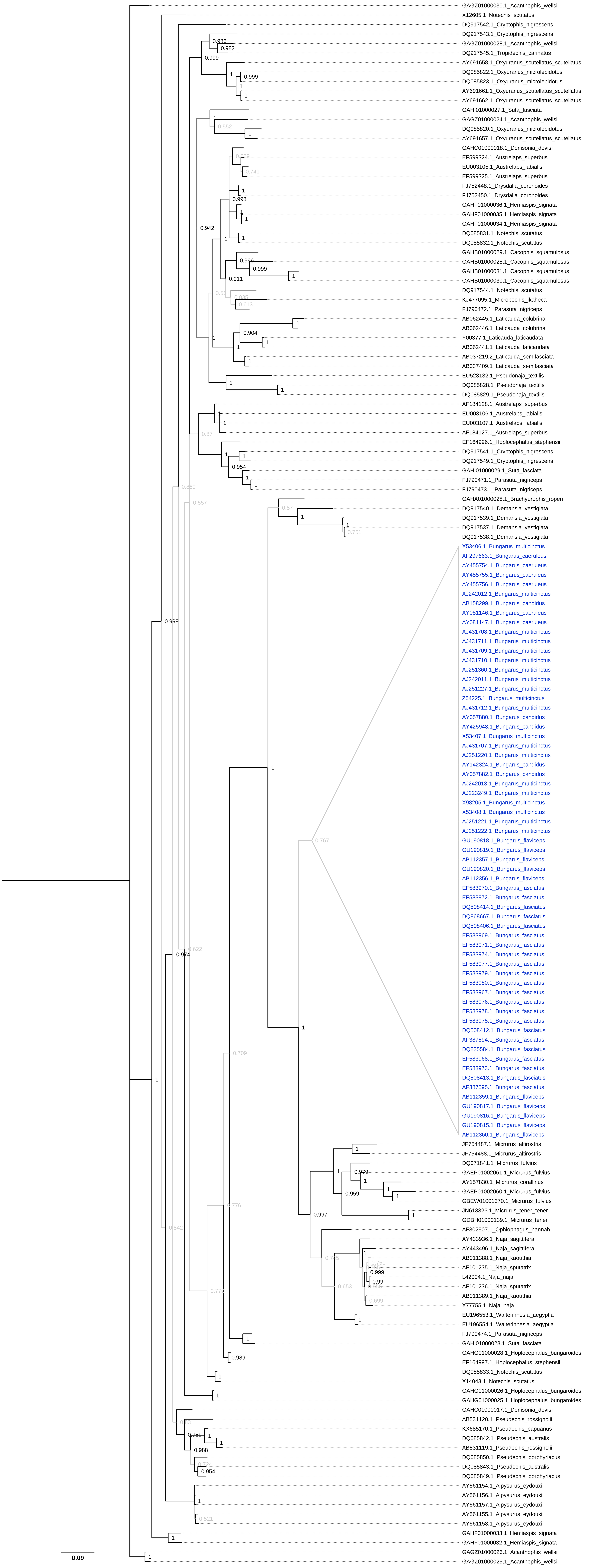

This figure depicts the phylogeny for β-bungarotoxins group with representatives from the other non-pancreatic group I PLA_2 sets. Branches with strong node supports (BPP ≥ 0.9) are shown as thick black lines.

Figure S7. Bayesian phylogenetic reconstruction for *Micrurus* group PLA_2s

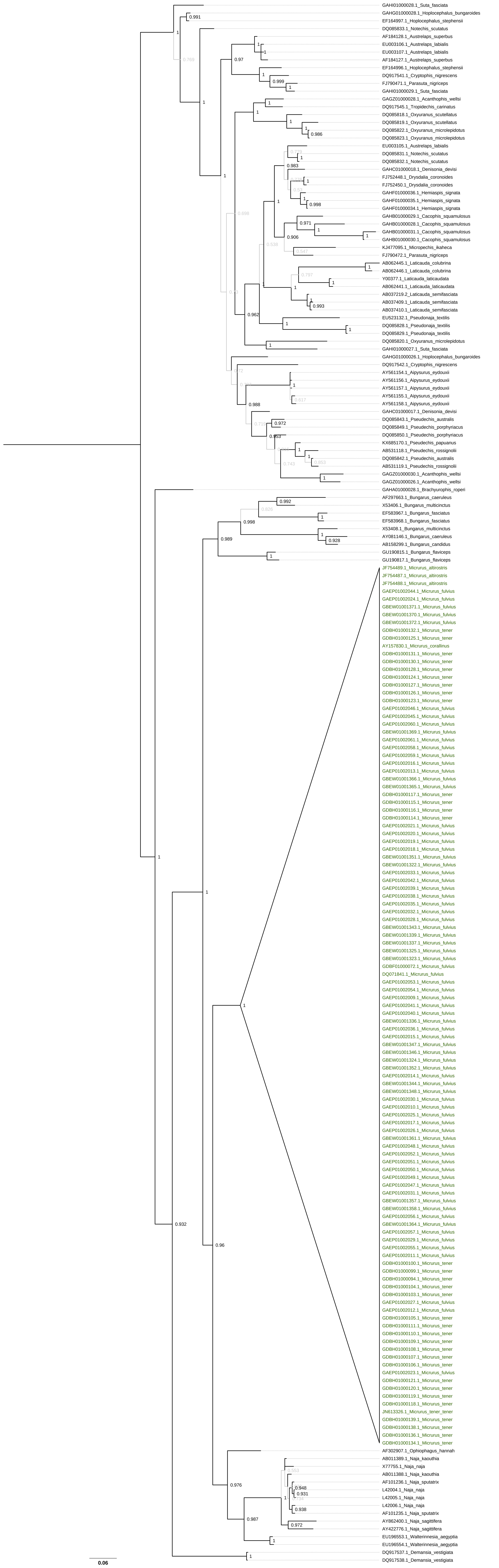

This figure depicts the phylogeny for the *Micrurus* group with representatives from the other non-pancreatic group I PLA_2 sets. Well-supported branches (BPP ≥ 0.9) are shown as thick black lines.

Figure S8. Bayesian phylogenetic reconstruction for *Naja* group PLA_2s

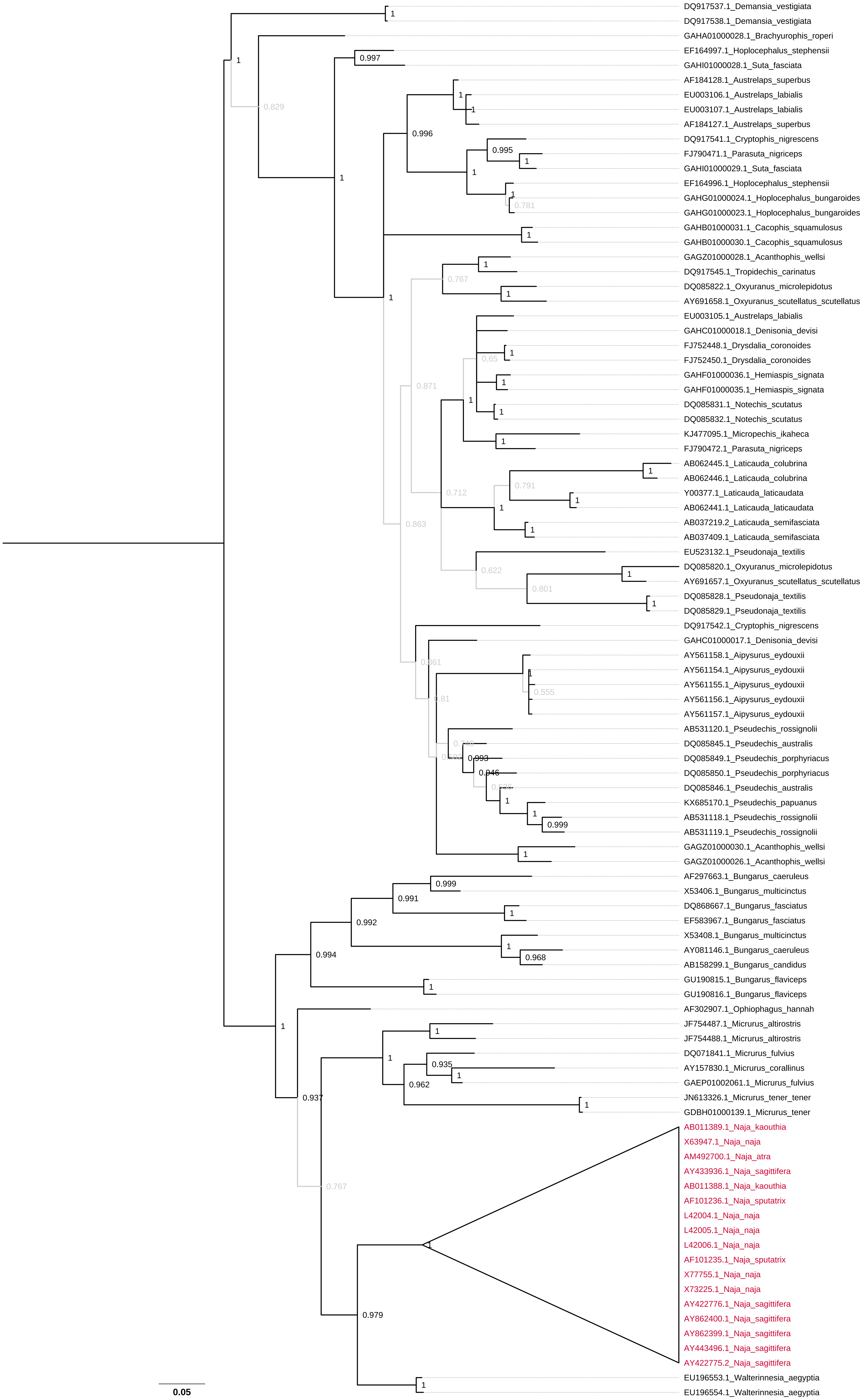

Phylogeny illustrating the evolutionary relationships of *Naja* group and other non-pancreatic group I PLA_2 sets is presented here. Well-supported branches (BPP ≥ 0.9) are shown as thick black lines.

Figure S9. Bayesian phylogenetic reconstruction for the sea krait PLA₂s

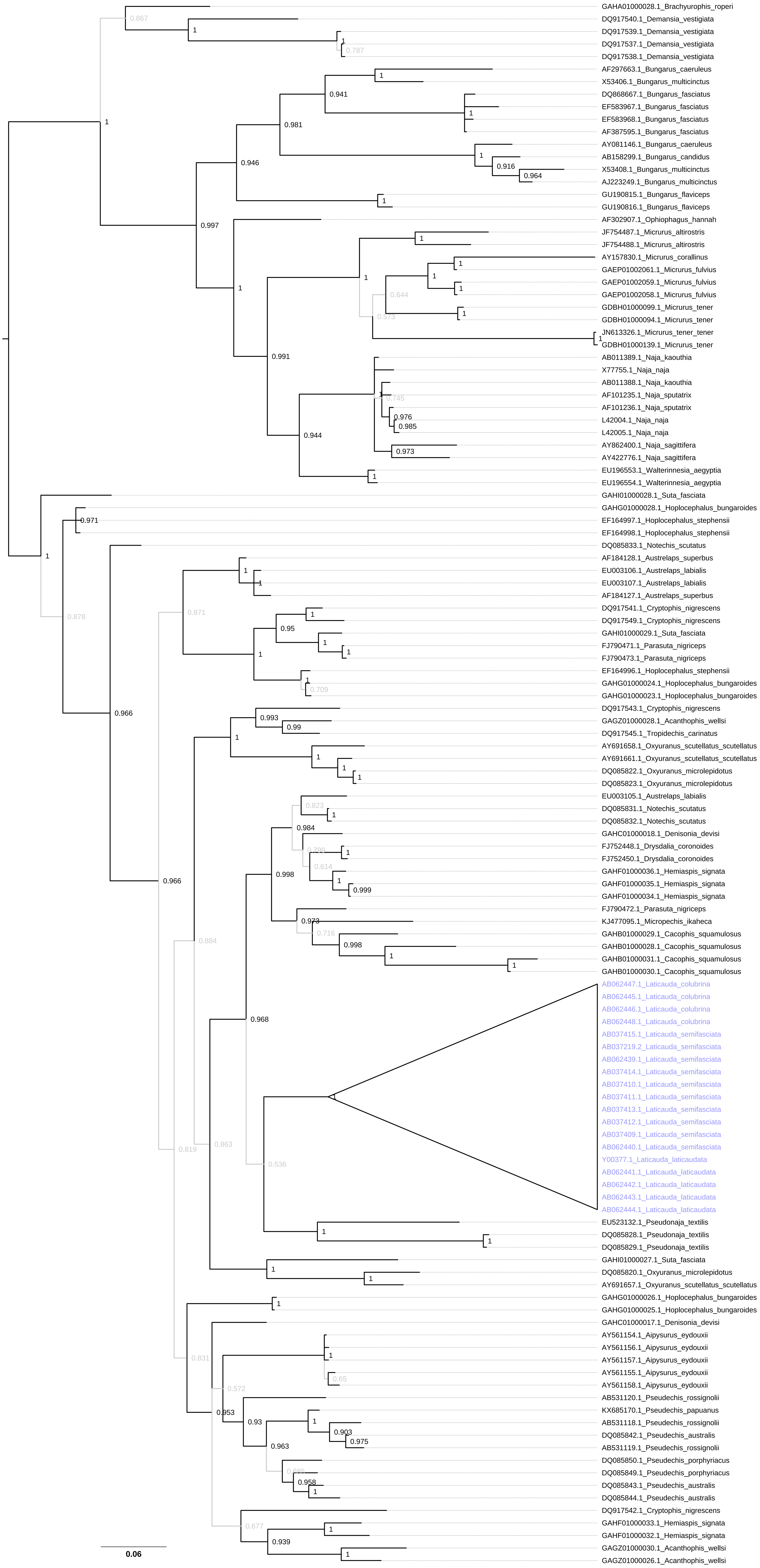

The figure presented here depicts the phylogeny of the sea krait group and other non-pancreatic group I PLA₂ sets. Thick lines indicate branches with BPP ≥ 0.9.

Figure S10. Bayesian phylogenetic reconstruction of group II PLA_2s (Viperidae)

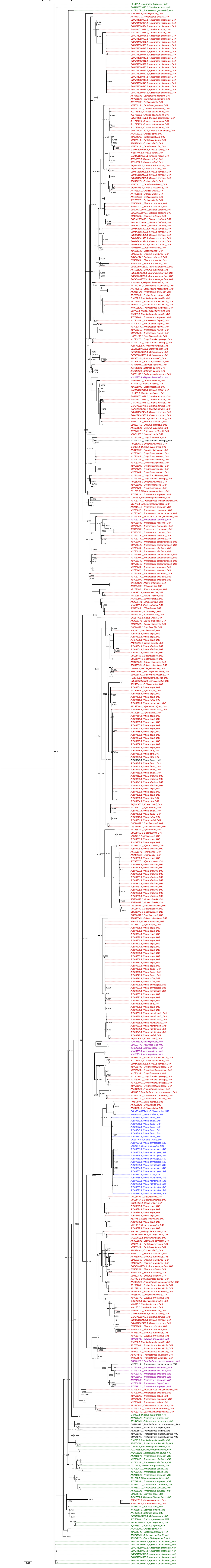

The presented phylogeny describes the evolutionary relationships of group II viperid PLA_2s. Subtypes are colour coded (D-, K-, N-, and S-type, with red, green, magenta and blue colour, respectively). Well-supported branches (BPP ≥ 0.9) are shown in thick black lines.
